# Antiangiogenic Effect of Ethanol Extract of *Vigna angularis* via Inhibition of Phosphorylation of VEGFR2, Erk, and Akt

**DOI:** 10.1155/2015/371368

**Published:** 2015-08-19

**Authors:** Oh Sung Kwon, Myoung Seok Jeong, Bonglee Kim, Sung-Hoon Kim

**Affiliations:** ^1^Department of East West Medical Science, Graduate School of East West Medical Science, Kyung Hee University, Suwon 446-701, Republic of Korea; ^2^College of Korean Medicine, Kyung Hee University, Seoul 130-701, Republic of Korea

## Abstract

Though dietary azuki bean (*Vigna angularis*) seed containing antioxidant proanthocyanidins was known to have multibiological activities including antioxidant, hypotensive, anti-inflammatory, and immunomodulatory activities, the antiangiogenic activity of ethanol extract of *Vigna angularis* (EVA) was never reported so far. In the present study, the antiangiogenic mechanism of EVA was examined in human umbilical vein endothelial cells (HUVECs). EVA showed weak cytotoxicity in HUVECs, while it significantly suppressed the VEGF induced proliferation of HUVECs. Consistently, wound healing assay revealed that EVA inhibited the VEGF induced migration of HUVECs. Also, EVA abrogated the VEGF induced tube formation of HUVECs in a concentration dependent fashion. Furthermore, Matrigel plug assay showed that EVA significantly reduced the hemoglobin level of Matrigel plug in mice compared to untreated control. Of note, EVA effectively attenuated the phosphorylation of VEGFR2, Erk, and Akt in VEGF-treated HUVECs. Overall, our findings suggest that EVA inhibits angiogenesis in VEGF-treated HUVECs via inhibition of phosphorylation of VEGFR2, ERK, and Akt.

## 1. Introduction 

Angiogenesis is well known as a phenomenon that makes new blood vessels from the preexisting vasculature [1]. The aberrant angiogenesis has role in a variety of diseases such as cancer [2, 3], psoriasis [4], arthritis [5], obesity [6], endometriosis [7], atherosclerosis [8], hypertension [9], diabetic retinopathy [10], and age-related macular degeneration [11]. Many of angiogenesis inhibitors have been developed for cancer treatment and prevention [12, 13], since Judah Folkman first claimed the critical role of angiogenesis for tumor growth [14].

Vascular endothelial growth factor (VEGF) binds to VEGF receptors (VEGFR), VEGFR1, VEGFR2, and VEGFR3, during angiogenesis [15]. Among these, VEGFR2 (KDR/Flk-1) has most important role in angiogenesis, including transduction of angiogenic signals after binding VEGF [16]. VEGFR2 became dimerized after the binding [17], promoting the activation of extracellular signal-regulated kinases (ERKs), phosphatidylinositol 3-kinase (PI3K), and Akt [18–20].

Recently angiogenesis inhibitors [21–24] from natural products are attractive, though not powerful, with less side effects such as fetal development, bleeding, blood clot, hypertension, and protein in urine [25, 26]. Our team also reported potent antiangiogenic materials from natural products, such as STB-HO [27], beta-sitosterol [28], cryptotanshinone [29], and emodin [30].

*Vigna angularis* that has been usually used as dietary azuki bean was known to have antioxidative [31], hepatoprotective [32], antimicrobial [33], osteogenic [34], anti-inflammatory [9, 35], hypotensive [36, 37], antimetastatic [38], and immunomodulatory [39] activities. Until now, there is no evidence on the antiangiogenic activity of* Vigna angularis*. We studied the antiangiogenic mechanism of ethanol extract of* Vigna angularis* (EVA) in VEGF-treated human umbilical vein endothelial cells (HUVECs) using cytotoxicity assay, proliferation assay, wound healing migration assay, tube formation assay, Matrigel plug assay, and Western blotting.

## 2. Materials and Methods

### 2.1. Preparation of Ethanol Extract of* Vigna angularis*

Azuki bean (*Vigna angularis*) was purchased from Hanil Herbal Company (Seoul, Republic of Korea), identified by Professor Namin Baek, Kyung Hee University, and stored at Cancer Preventive Material Development Research Center (CPMDRC), Kyung Hee University, Republic of Korea, with voucher specimen number (2012-05-07). Azuki bean (600 g) was socked in 95% ethanol for one week at room temperature. The EtOH solution was then filtered, evaporated under reduced pressure, and lyophilized to obtain 14.87 g of ethanol extract of* Vigna angularis* (EVA yield = 2.47%). The extract was dissolved in DMSO.

### 2.2. Cell Culture

Human umbilical vein endothelial cells (HUVECs, passages between 4 and 6) were obtained from fresh human umbilical cord veins after collagenase treatment as described previously [22, 40]. The cells were maintained in M199 (Invitrogen, Carlsbad, CA) with 20% fetal bovine serum (FBS), 5 U/mL heparin and 3 ng/mL basic fibroblast growth factor (R&D Systems, Minneapolis, MN), and 100 U/mL of antibiotic-antimycotic in 0.1% gelatin coated flasks. The cells were cultured at 37°C in a humidified atmosphere containing 5% CO_2_.

### 2.3. Cytotoxicity Assay

Cytotoxicity induced by EVA was evaluated in HUVECs by 3-(4,5-dimethylthiazol-2-yl)-2,5-diphenyl tetrazolium bromide (MTT) assay. The cells were seeded onto microplates (1 × 10^4^ cells/well) and treated with 0, 10, 20, 30, 40, and 50 *μ*g/mL of EVA for 48 h. The cells were treated with MTT solution (1 mg/mL) for 2 h. DMSO was added to cells for 2 h. Optical density (OD) of the solution was measured using a microplate reader (Molecular Devices Co., Sunnyvale, CA) at 570 nm. Cell viability was calculated as a percentage of viable cells in EVA-treated group versus untreated control by the following equation. Cell viability (%) = [OD (EVA) − OD (Blank)]/[OD (Control) − OD (Blank)] × 100.

### 2.4. Proliferation Assay

Cell proliferation was determined using a 5-bromo-2′-deoxyuridine (BrdU) colorimetric assay kit (Roche, Mannheim, Germany). Briefly, HUVECs (5 × 10^3^ cells/well) were seeded into 0.1% gelatin coated microplates and incubated in a humidified atmosphere containing 5% CO_2_ at 37°C. After starvation for 6 h in M199 containing 5% heat-inactivated FBS, the cells were treated with various concentrations of EVA (10, 20, and 30 *μ*g/mL) in the presence or absence of VEGF (50 ng/mL) and incubated at 37°C for 48 h. 10 *μ*L of BrdU (100 *μ*g/mL) was added; the cells were incubated at 37°C for 6 h. The cells were fixed with anti-BrdU and measured by the substrate reaction. 25 *μ*L of 1 M H_2_SO_4_ was added to stop the reaction and the absorbance was calculated by using an ELISA reader (Molecular Devices Co., USA) (450 nm with 690 nm correction).

### 2.5. Wound Healing Migration Assay

The migratory activity of HUVECs was tested by a wound healing assay. The cells (4 × 10^5^ cells/2 mL) were exposed to various concentrations of EVA (10 and 20 *μ*g/mL) in a 6-well plate in the presence or absence of VEGF (50 ng/mL) and incubated in a humidified atmosphere containing 5% CO_2_ at 37°C. The plates were scratched with a 200 *μ*L pipet tip and washed with PBS for 3 times. Then, cells were treated with EVA extracts (0, 10, and 20 *μ*g/mL) in media. The cells were fixed and stained with Diff-Quick after 24 h, and randomly chosen fields were photographed with a fluorescence microscope (AXIO observer A1, Zeiss, Germany). The migrated cells were calculated.

### 2.6. Tube Formation Assay

Tube formation assay was conducted as described previously [41]. 250 *μ*L of Matrigel (Becton Dickinson Labware, Bedford, MA) was added to 24-well plates and incubated at 37°C for 1 h. HUVECs (1 × 10^5^ cells/well) were treated with EVA (0, 10, and 20 *μ*g/mL) in the presence or absence of VEGF (50 ng/mL). And cells were treated with LY294002 (20 *μ*M), PD98059 (50 *μ*M), EVA (20 *μ*g/mL), and VEGF (50 ng/mL). Randomly chosen fields were photographed with Axiovert S 100 light microscope (Carl Zeiss, USA) at 100x magnification after 18 h. Tube network was measured by using NIH Scion image program.

### 2.7. Matrigel Plug Assay

Six-week-old C57BL/6 mice (Chungang animal expt. Co., Seoul, Republic of Korea) were given subcutaneous injection of 0.5 mL of growth factor reduced Matrigel containing VEGF (50 ng/mL) and heparin (10 U/mL). EVA extracts (40 *μ*g/mL in PBS) were orally administrated (300 *μ*g/kg) after 7 days of Matrigel injection (three mice per group). Ten days later, the mice were sacrificed, and the matrigel plugs were detached. To indirectly quantify the formation of new blood vessel, the amount of hemoglobin (Hb) was calculated using the hemoglobin assay kit (YD Diagnostics, Republic of Korea). The concentration of Hb was measured from standard curve made from the known amount of Hb provided according to the manufacturer's protocol.

### 2.8. Western Blotting

After treatment of EVA (0, 10, or 20 *μ*g/mL) in a presence of VEGF (50 ng/mL), HUVECs were lysed with RIPA buffer (50 mM Tris-HCl, 150 mM NaCl, 1% NP-40, 0.25% sodium deoxycholic acid, 1 M EDTA, 1 mM Na_3_VO_4_, 1 mM NaF, and protease inhibitors cocktail, pH 7.4). Samples were quantified by using a Bio-Rad DC protein assay kit II (Bio-Rad, Hercules, CA, USA), separated by electrophoresis on 8 to 15% SDS-PAGE gel and electrotransferred onto a Hybond ECL transfer membrane (Amersham Pharmacia, Piscataway, NJ, USA). After blocking with 3–5% nonfat skim milk, the membrane was incubated with antibodies for p-Akt, Akt, VEGFR2 (FLK1), p-Erk, and Erk (Cell Signaling Technology, Danvers, MA) and *β*-actin (Sigma Aldrich Co., St. Louis, MO). Blots were exposed to horseradish peroxidase- (HRP-) conjugated secondary anti-mouse or rabbit antibodies (AbDserotec, kidlington, UK). Protein expression was measured by using enhanced chemiluminescence (ECL) system (Amersham Pharmacia, Piscataway, NJ, USA).

### 2.9. Statistical Analyses

All data were expressed as mean ± SD (standard deviation). The statistically significant differences between untreated control and EVA-treated cells were calculated by ANOVA test followed by a post hoc analysis (Tukey *t*- or Dunnettes multiple comparison test) using Prism software 5 (GraphPad Software, Inc., San Diego, CA, USA).

## 3. Results

### 3.1. EVA Shows Weak Cytotoxicity in HUVECs and Suppressed VEGF Induced Proliferation of HUVECs

**Figure 1:**
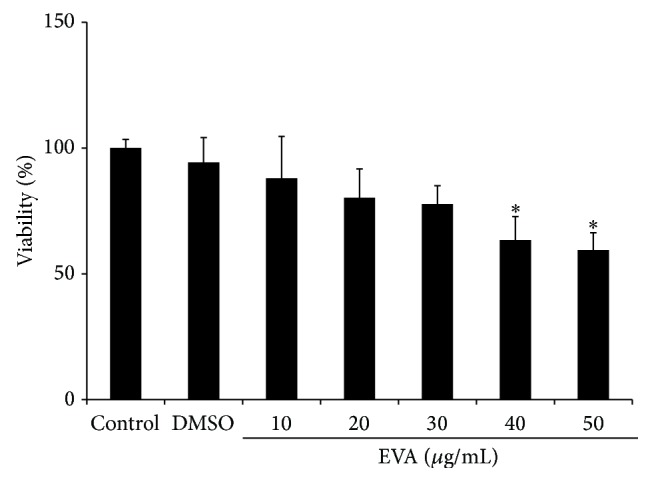
EVA exerts weak cytotoxicity against HUVECs. Cytotoxic effect of EVA was evaluated in HUVECs by MTT assay. The cells were seeded onto 96-well microplates at a density of 1 × 10^4^ cells/well and treated with various concentrations of EVA (0, 10, 20, 30, 40, and 50 *μ*g/mL) for 48 h. Data are presented as means ± SD. ^*∗*^ *p* < 0.05 versus DMSO-treated group.

**Figure 2:**
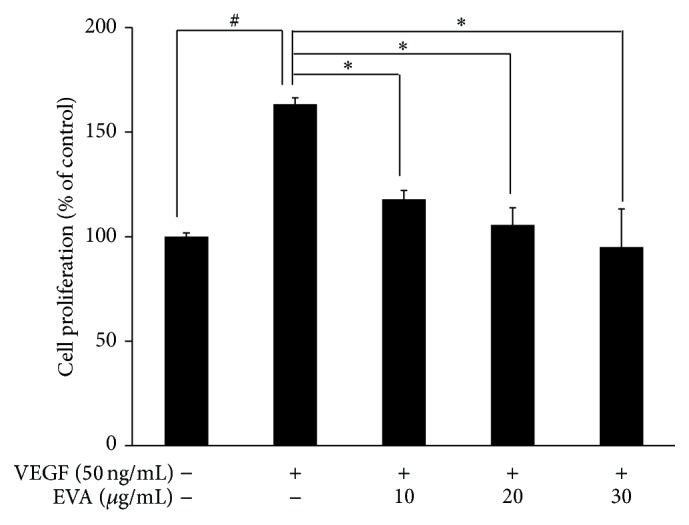
EVA inhibits VEGF induced proliferation in HUVECs. Cell proliferation was determined using a BrdU colorimetric assay kit. Cells (5 × 10^3^ cells/well) were seeded onto 96-well plates and incubated for 24 h. After 6 h starvation, the cells were exposed to various concentrations of EVA (10, 20, and 30 *μ*g/mL) in the presence or absence of VEGF (50 ng/mL) and incubated for 48 h at 37°C. Data are presented as means ± SD. ^*∗*, #^ *p* < 0.05 versus VEGF only treated group.

Cytotoxicity of EVA was evaluated in HUVECs using MTT assay. As shown in Figure 1, EVA decreased the viability of HUVECs to ~60% at the concentration of 50 *µ*M for 48 h. EVA treatment showed cytotoxicity in VEGF-treated HUVECs in a concentration dependent manner (Figure 2).

### 3.2. EVA Significantly Inhibits the VEGF Induced Migration and Tube Formation in HUVECs

It was well known that endothelial cells migrate in tandem using adhesion molecules such as integrins and form loops to become a full-fledged vessel lumen like tube formation [42]. To check the physiological angiogenic formation, wound healing assay and tube formation assay were carried out in HUVECs.

**Figure 3:**
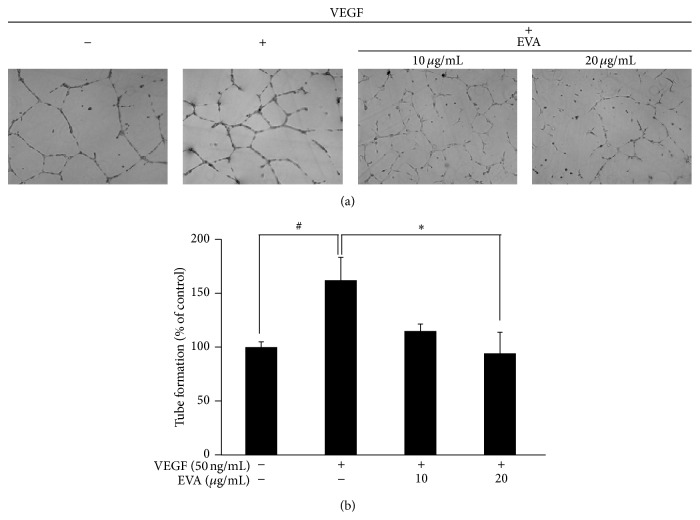
EVA abrogates VEGF induced tube formation in HUVECs. (a) Matrigel (250 *μ*L) was added to 24-well plates and allowed to solidify for 1 h at 37°C. HUVECs (1 × 10^5^ cells/well) were treated with 10 and 20 *μ*g/mL of EVA in the presence or absence of VEGF (50 ng/mL) and incubated at 37°C. After 18 h, randomly chosen fields were photographed under an Axiovert S 100 light microscope. (b) Bar graph represents the quantification of tube formation of untreated control or VEGF-treated control and EVA-treated groups. Data are presented as means ± SD. ^*∗*, #^ *p* < 0.05 versus VEGF-treated group.

**Figure 4:**
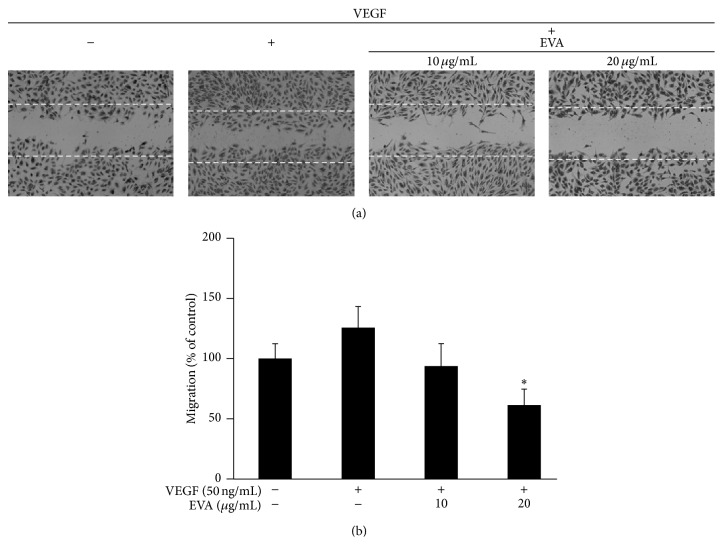
EVA suppresses VEGF induced migration in HUVECs by wound healing assay. (a) The cells (4 × 10^5^ cells/2 mL) were treated with 0, 10, and 20 *μ*g/mL of EVA in a 6-well plate in the presence of VEGF (50 ng/mL) and incubated at 37°C. The number of cells migrated into the scratched area was calculated. (b) Bar graph represents the quantification of migrated cells of untreated control or VEGF-treated control and EVA-treated groups. Data are presented as means ± SD. ^*∗*^ *p* < 0.05 versus VEGF only treated group.

As shown in Figures 3(a) and 3(b), EVA significantly reduced the number of tube forming endothelial cells, whereas tube-like formation by endothelial cells was enhanced by VEGF in HUVECs. Similarly, EVA significantly suppressed the VEGF induced migratory activity of HUVECs, while the gap physically made by tip was narrowed via repair through migratory activity of VEGF-treated HUVECs (Figures 4(a) and 4(b)).

### 3.3. EVA Significantly Reduces Hemoglobin Content in VEGF Induced Angiogenesis of Matrigel Plugs of C57BL/6 Mice

**Figure 5:**
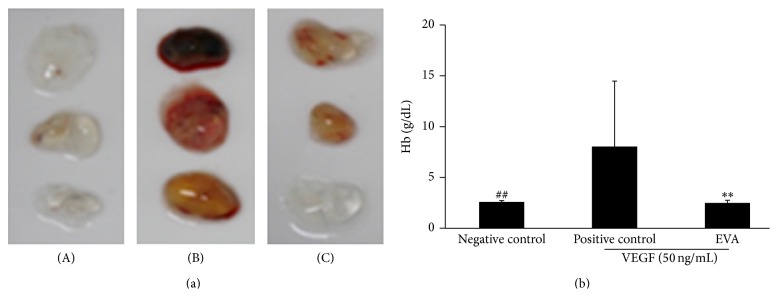
EVA significantly reduces hemoglobin content in VEGF induced angiogenesis in Matrigel plugs of mice. Six-week-old C57BL/6 mice were given EVA (300 *μ*g/kg) extracts via oral administration for 7 days before growth factor reduced Matrigel with VEGF (25 ng) and heparin (5 U) was subcutaneously injected into right flank of C57BL/6. Ten days later, Matrigel plugs were removed. (a) The amount of hemoglobin (Hb) was measured using the hemoglobin assay kit. ((A), (B), and (C) represent negative control, positive control, and EVA-treated group, resp.) (b) Bar graph represents the quantification of Hb concentration from each of groups. Data are presented as means ± SD. ^*∗∗*, ##^ *p* < 0.01 versus positive control group.

To confirm the antiangiogenic activity of EVA, Matrigel plug assay was carried out. EVA (300 *μ*g/kg) extract was administered orally to C57BL/6 mice for 7 days before growth factor reduced Matrigel was subcutaneously injected into right flank of mice. As shown in Figures 5(a) and 5(b), EVA significantly reduced hemoglobin content in VEGF induced angiogenesis of Matrigel plugs of C57BL/6 mice, while dark red color was shown in Matrigel plugs of untreated control group.

### 3.4. EVA Effectively Attenuates the Phosphorylation of VEGFR2, Erk, and Akt in VEGF-Treated HUVECs

**Figure 6:**
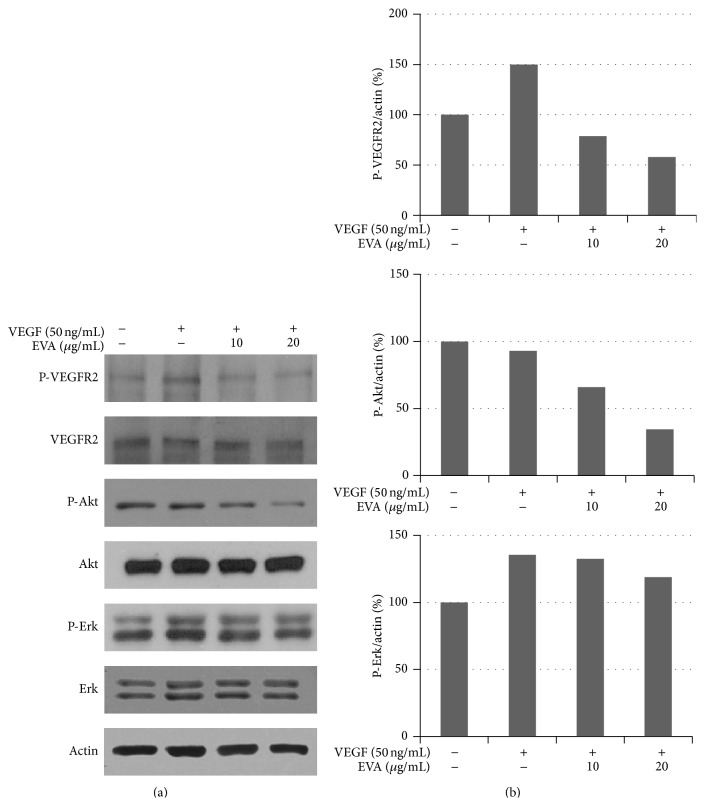
EVA effectively attenuates the phosphorylation of VEGFR2, Erk, and Akt in VEGF-treated HUVECs. Cells were treated with 10 or 20 *μ*g/mL of EVA in the presence of VEGF for 20 h and lysed in RIPA buffer. (a) The cell lysates were prepared and subjected to Western blotting for p-VEGFR2, VEGFR2, p-Akt, Akt, p-Erk, Erk, and *β*-actin. (b) Bar graphs represent fold change between actin and p-VEGFR2, p-Akt, or p-Erk groups.

Since Dr. Judah Folkman suggested the importance of tumor angiogenesis, a variety of angiogenesis related factors such as VEGF, bFGF, PDGF, angiopoietin, and transforming growth factor-*β* (TGF-*β*) were studied for years [1, 43]. To elucidate antiangiogenic molecular mechanism of EVA, Western blot analysis was performed in VEGF-treated HUVECs. As shown in Figure 6, EVA effectively attenuated the phosphorylation of VEGFR2 and Erk and Akt in VEGF-treated HUVECs.

### 3.5. Akt, Erk Inhibitors, and EVA Attenuate Tube Formation Enhanced by VEGF in HUVECs

**Figure 7:**
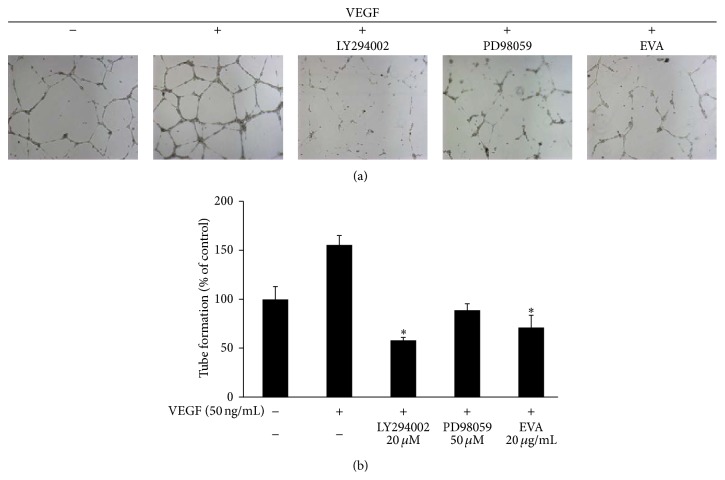
Akt, Erk inhibitors, and EVA attenuate tube formation enhanced by VEGF in HUVECs. (a) Matrigel (250 *μ*L) was added to 24-well plates and EVA (20 *μ*g/mL), LY294002 (Akt inhibitor, 20 *μ*M), and PD98059 (Erk inhibitor, 50 *μ*M) were treated to HUVECs (1 × 10^5^ cells/well) in the presence of VEGF (50 ng/mL) and incubated at 37°C. After 18 h, randomly chosen fields were photographed under an Axiovert S 100 light microscope. (b) Bar graph represents the quantification of tube formation of each group. Data are presented as means ± SD. ^*∗*^ *p* < 0.05 versus VEGF-treated group.

To investigate the roles of Erk and Akt in angiogenesis, tube formation assay was conducted with HUVECs in the presence of LY294002 (Akt inhibitor) or PD98059 (Erk inhibitor). As shown in Figures 7(a) and 7(b), Akt, Erk inhibitors, and EVA treatments inhibited tube formation enhanced by VEGF. Particularly, LY294002 and EVA significantly attenuated the tube formation.

## 4. Discussion

Azuki bean (*Vigna angularis*) has been used in east Asian food, and its coat of seed was known to contain water-soluble anthocyanins, catechins, and flavonols [44]. Despite multibiological activities of azuki bean, its antiangiogenic activity was never reported until now. So, this study elucidates the antiangiogenic mechanism of azuki bean in VEGF-treated HUVECs.

Though EVA exerted weak cytotoxicity in HUVECs, EVA significantly suppressed the VEGF induced proliferation of HUVECs at nontoxic concentrations, implying that EVA has antiangiogenic potential by blocking pathological angiogenic process via VEGF induced proliferation of endothelial cells, since these endothelial cells become motile and invasive and protrude filopodia, which drives new vessel formation [14, 25, 42].

Also, EVA inhibited VEGF induced migration of HUVECs in wound healing assay and suppressed VEGF induced tube formation of HUVECs in a concentration dependent manner in tube formation assay, strongly indicating that EVA could inhibit the motility and tube formation of proliferative endothelial cells.

To confirm* in vivo* antiangiogenic activity of EVA, Matrigel plug assay was performed in C57BL6 mice orally administered by EVA extracts for 1 week earlier. Here, we observed less red color in Matrigel plugs isolated from mice treated by EVA, while black red color was shown in those of untreated control group. The treatment of EVA significantly reduced the hemoglobin level of Matrigel plug in mice compared to untreated control, demonstrating antiangiogenic activity of EVA. However, it requires further* in vivo* angiogenesis studies using Matrigel plug assay via direct EVA treatment in the increased number of mice or CAM assay in the near future.

It was well documented that several molecules were involved in angiogenesis process. Angiogenesis is processed via the interaction of the VEGF family of proangiogenic cytokines and their receptors. The VEGF family includes VEGFA, VEGFB, VEGFC, VEGFD, VEGFE, and placental growth factor (PGF) with three receptors including VEGFR1 (Fms-like tyrosine kinase 1/Flt-1), VEGFR2 (Flk-1/KDR), and VEGFR3 (Flt-4) [20, 45, 46]. Also, VEGFR2 expression is typically limited to vessel endothelial cells, whereas VEGFR1 can be usually found in bone-marrow derived progenitors [47]. Furthermore, there was evidence that Akt and Erk mediate angiogenesis in endothelial cells [48–53]. sc-221226, an Akt inhibitor, attenuated sphingosine-1-phosphate induced angiogenesis in endothelial progenitor cells [54]. Also, low molecular weight fucoidan inhibited angiogenesis* in vitro* and* in vivo* evidenced by attenuation of tube formation of hypoxic HUVECs and suppressing Akt activity reduced hypoxia-induced VEGF secretion in T24 cells, supporting the involvement of Akt in the induction of HIF-1*α* and VEGF and angiogenesis [55]. Epidermal growth factor-like domain 7 promotes angiogenesis via ERK signaling pathway in endothelial cells [56]. And fractalkine, a large cytokine protein of 373 amino acids, stimulates angiogenesis by activation of ERK and Akt [57]. These results support our conclusions. The result of Western blotting revealed that EVA effectively attenuated the phosphorylation of VEGFR2, Erk, and Akt in VEGF-treated HUVECs, implying that EVA inhibited angiogenesis in VEGF-treated HUVECs via suppression of VEGFR2, Akt, and Erk. Also, LY294002 (Akt inhibitor) showed similar effect on VEGF-enhanced tube formation, indicating that inhibitory effect of EVA on angiogenesis in HUVECs could be by inhibition of Akt. In addition, azuki bean was reported to contain four furanylmethyl glycosides, angularides A–D, one ent-kaurane diterpene glycoside, angularin A, and four triterpenoid saponins, angulasaponins A–D, cyanidin and (+)-catechin, proanthocyanidins, digalactosyl ononitol, and gibberellin 2-oxidases [36, 44, 58–60]. Among them, anthocyanidin was well known to exert antiangiogenic activity in endothelial cells [61, 62].

In summary, EVA significantly suppressed the VEGF induced proliferation, migration, tube formation, and angiogenesis in HUVECs, and the molecular mechanism is the phosphorylation inhibition of VEGFR2 and Erk and Akt in VEGF-treated HUVECs. Overall, our findings suggest that EVA inhibits angiogenesis in VEGF-treated HUVECs via phosphorylation inhibition of VEGFR2 and Erk and Akt. Our study suggests that EVA is a promising natural agent which has antiangiogenic activity in HUVECs. However, further study is required to find out a leading antiangiogenic constituent from EVA and its potency for clinical application as a functional food.
